# The Circular RNA Circ-ANAPC7 as a Biomarker for the Risk Stratification of Myelodysplastic Syndrome

**DOI:** 10.1007/s12288-022-01594-2

**Published:** 2022-11-18

**Authors:** Fang Zhou, Shuying Zhang, Mei Huo, Ying Zhou, Libo Jiang, Hong Zhou, Ying Qu

**Affiliations:** 1 Department of Laboratory Diagnosis, The Second Affiliated Hospital of Qiqihar Medical College, No. 37, Zhong Huaxi Road, Qiqihar, Heilongjiang China; 2 Department of Hematology, The Second Affiliated Hospital of Qiqihar Medical College, Qiqihar, China

**Keywords:** Myelodysplastic syndrome, Risk stratification, Circ-ANAPC7, Biomarker

## Abstract

To assess the diagnostic value of circ-ANAPC7 expression levels in MDS and its risk stratification. This is a retrospective observational study. This study enrolled 125 patients diagnosed with MDS and divided them into five groups according to IPSS-R (very high group, 25; high group, 25; intermediate group, 25; low group, 25; and very low group, 25), and 25 patients with IDA were studied as control group from our bone marrow cell bank. Bone marrow cell were used as material in this study to measure the expression level of circ-ANAPC7 by qRT-PCR. An evaluation of diagnostic value was conducted using ROC curves. Circ-ANAPC7 expression levels were 5.623 ± 4.483, 28.396 ± 12.938, 91.867 ± 37.010, 202.525 ± 54.911, 337.633 ± 86.013, and 502.269 ± 98.410 from the control group to the very high group, respectively (*p* < 0.05). Circ-ANAPC7 expression was gradually upregulated with the risk stratification of MDS. The AUCs of circ-ANAPC7 were 0.973, 0.996, 0.951, 0.920, and 0.907 in the control group/very low group, very low group/low group, low group/intermediate group, intermediate group/high group, and high group/very high group, respectively. In this study, the expression level of circ-ANAPC7 was found to be a promising biomarker for MDS. It may be added to the scoring system to better identify risk groups.

## Introduction

Myelodysplastic syndromes (MDSs) are heterogeneous clonal diseases caused by haematopoietic stem cell disorders characterized by peripheral blood cytopenia, ineffective hemopoiesis, and risk of progression to acute myeloid leukaemia (AML) [1]. Age is a significant factor in the occurrence of MDS [2]. Approximately 71.5 years have been reported as the median age at diagnosis in studies [3]. Cytopenia and poor prognosis of MDS make it a serious disease with a significant impact on everyday life quality [4]. Currently, there are several useful and powerful tools to determine prognosis in MDS, including the International Prognostic Scoring System (IPSS) and the Revision International Prognostic Scoring System (IPSS-R). [5]. However, some shortcomings have also been identified in clinical application. Therefore, the exploration of the pathogenesis and risk stratification determination of MDS has become a research hotspot in recent years.

It has become common practice to use second-generation gene sequencing in clinical practice due to the rapid development of gene technology. Although the molecular mechanism of MDS has not been fully clarified, current studies have found that MDS is mainly caused by epigenetic disorders and spliceosome dysfunction [6]. An epigenetic regulatory mechanism known as noncoding RNA (ncRNA) may contribute to the occurrence and development of MDS [7]. Therefore, circular RNA (circRNA), a newly recognized special type of ncRNA formed by reverse splicing, is a rising star of the RNA family, following microRNA (miRNA), with tremendous development potential [8, 9]. There is growing evidence that CircRNAs play a crucial role in regulating the progression of malignancies, such as glioma, hepatocellular carcinoma, and AML [10–12].

The circular RNA circ-ANAPC7 is one of the circRNA family and may play a role in AML pathogenesis [13]. Studies show that MDS and secondary AML cells exhibit similar mutations in many genes and functional categories, confirming that they are a disease continuum [14]. It is reasonable to speculate that circ-ANAPC7 may also play a certain regulatory role in the pathogenesis of MDS and may become a diagnostic marker of MDS.

This study aims to detect the expression level of circ-ANAPC7 in MDS, judge its diagnostic value in MDS, and compare whether there is any difference in circ-ANAPC7 in each risk stratification of MDS to provide a good indicator for the risk stratification of MDS.

## Materials and Methods

### Collection of Specimens

This is a retrospective observational study. This study enrolled 125 patients diagnosed with MDS and divided them into five groups according to IPSS-R (very high group, 25; high group, 25; intermediate group, 25; low group, 25; and very low group, 25), and 25 patients with IDA were studied as control group from our bone marrow cell bank. MDS was diagnosed according to WHO standards. All individuals were recruited from the Second Affiliated Hospital of Qiqihar Medical College. Chemotherapy had never been administered to any of the patients before. In all cases, bone marrow aspiration was used to obtain the specimens Mononuclear cells were isolated from bone marrow and immediately preserved at − 80 °C until needed. Ethics approval was obtained from the Second Affiliated Hospital of Qiqihar University for this study. It was the responsibility of all patients to provide written informed consent.

### Quantitative Real-Time PCR (qRT‒PCR)

The manufacturer's instructions were followed for RNA extraction using Trizol reagent. NanoDrop ND5000 (BioTeke Corporation, China) was used to determine the purity and concentration of RNA samples at 260 nm and 280 nm. Afterwards, the RevertAid First Strand cDNA Synthesis Kit (Fermentas, USA) was used to reverse-transcribe the RNA into cDNA. Finally, PCR was performed on an Aria Mx Real-Time PCR System (Agilent, USA) with SYBR Green PCR Master Mix (ABI, USA). For data analysis, using β-actin as a control, the endogenous value was determined, And the 2^−ΔΔCt^ method was used to calculate data. The sequence primers were as follows: hsa-circRNA-101141, F: 5′-GGGAGCAGCACTTAGGAACAT-3′, R: 5′-AAAGCTGGTACTTCTGAGGTGG-3′.

### Statistical Analysis

An analysis of all statistical data was conducted using SPSS 26.0 (United States). Variables with continuous distributions are expressed as means ± SDs or medians with interquartile ranges, while variables with categorical distributions are expressed as percentages. Shapiro–Wilk tests were used to evaluate normal distributions. In order to determine whether the variances in the six groups were homogeneous, the Levene test was used. Using the Games Howell test, comparisons were made among the six groups. An analysis of qualitative variables was conducted using the chi-square text. The circ-ANAPC7’s diagnostic value was assessed by the receiver operating characteristic (ROC) curve; the Youden index (specificity + sensitivity − 1) was used to calculate the cut-off value for circ-ANAPC7. If P values < 0.05, it was deemed statistically significant.

## Result

### Circ-ANAPC7 Expression is Upregulated in MDS

**Fig. 1:**
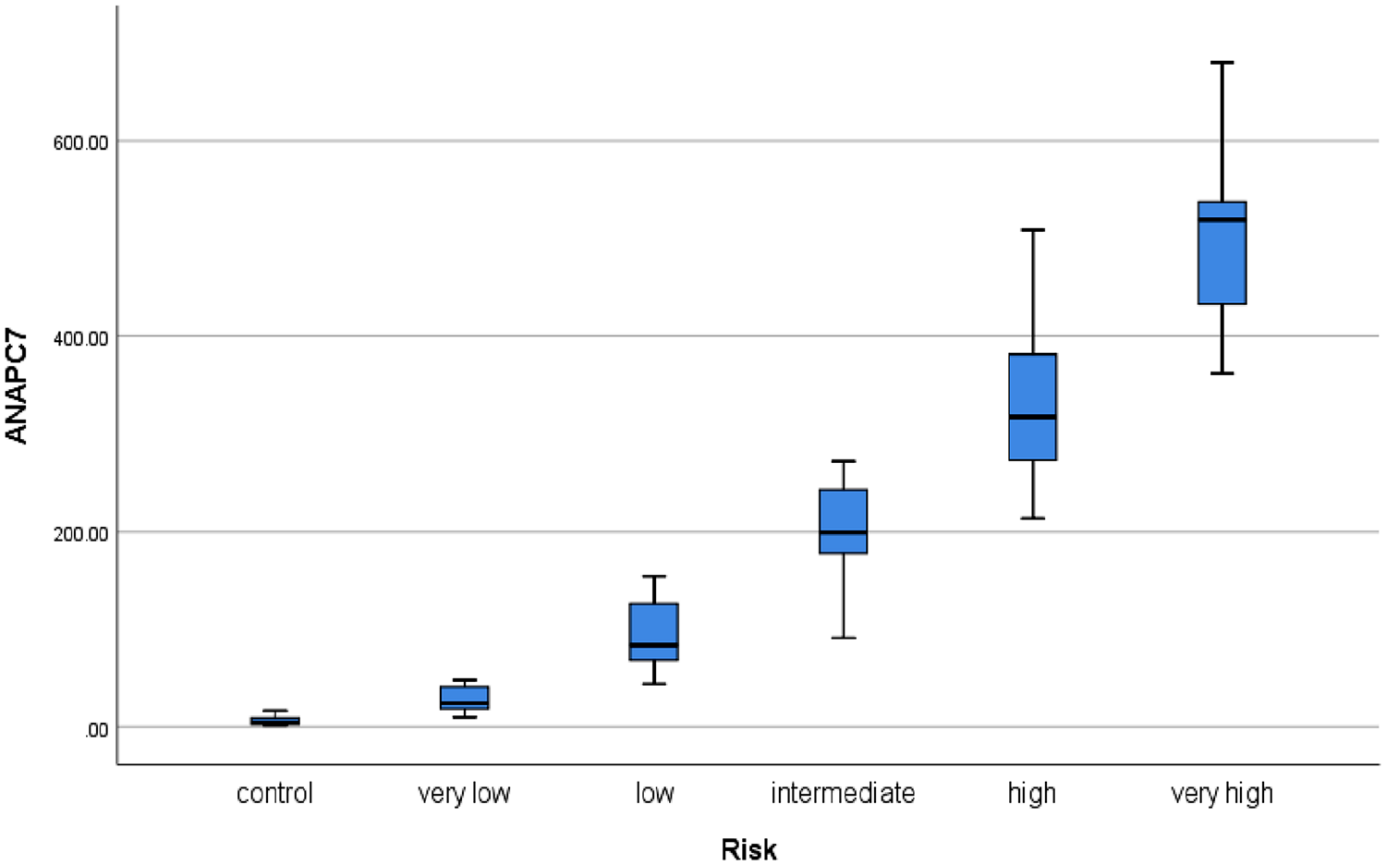
Expression level of circ-ANAPC7 was measured by qRT-PCR. Circ-ANAPC7 was significantly upregulated in MDS and gradually increased with the risk stratification of MDS

In Table 1, we describe the general characteristics of the subjects. Patients in various groups had similar ages and sexes. Circ-ANAPC7 expression levels were 5.623 ± 4.483, 28.396 ± 12.938, 91.867 ± 37.010, 202.525 ± 54.911, 337.633 ± 86.013, and 502.269 ± 98.410 from the control group to the very high group, respectively (Table 1). The difference was statistically significant between every two groups. Moreover, circ-ANAPC7 expression was gradually upregulated with the risk stratification of MDS (Fig. 1).

**Table 1:** Characteristics of subjects in each risk stratification group and the control group

	Control group n = 25	Very low group n = 25	Low group n = 25	Intermediate group n = 25	High group n = 25	Very high group n = 25
Age (years)	60.7 ± 13.3	66.3 ± 13.5	59.0 ± 10.5	54.3 ± 11.3	58 ± 12.5	63.7 ± 13.3
Sex, female (%)	6(24%)	8(32%)	9 (36%)	6(24%)	7 (28%)	8 (32%)
circ-ANAPC7	5.62 ± 4.48	28.40 ± 12.94	91.87 ± 37.01	202.53 ± 54.91	337.63 ± 86.01	502.27 ± 98.41
Range	< 12.16	12.16 ~ 43.56	43.56 ~ 149.11	149.11 ~ 279.25	279.25 ~ 399.01	> 399.01

Results are expressed as frequencies, means ± SDs

### Diagnostic Value of Circ-ANAPC7 in MDS

**Fig. 2:**
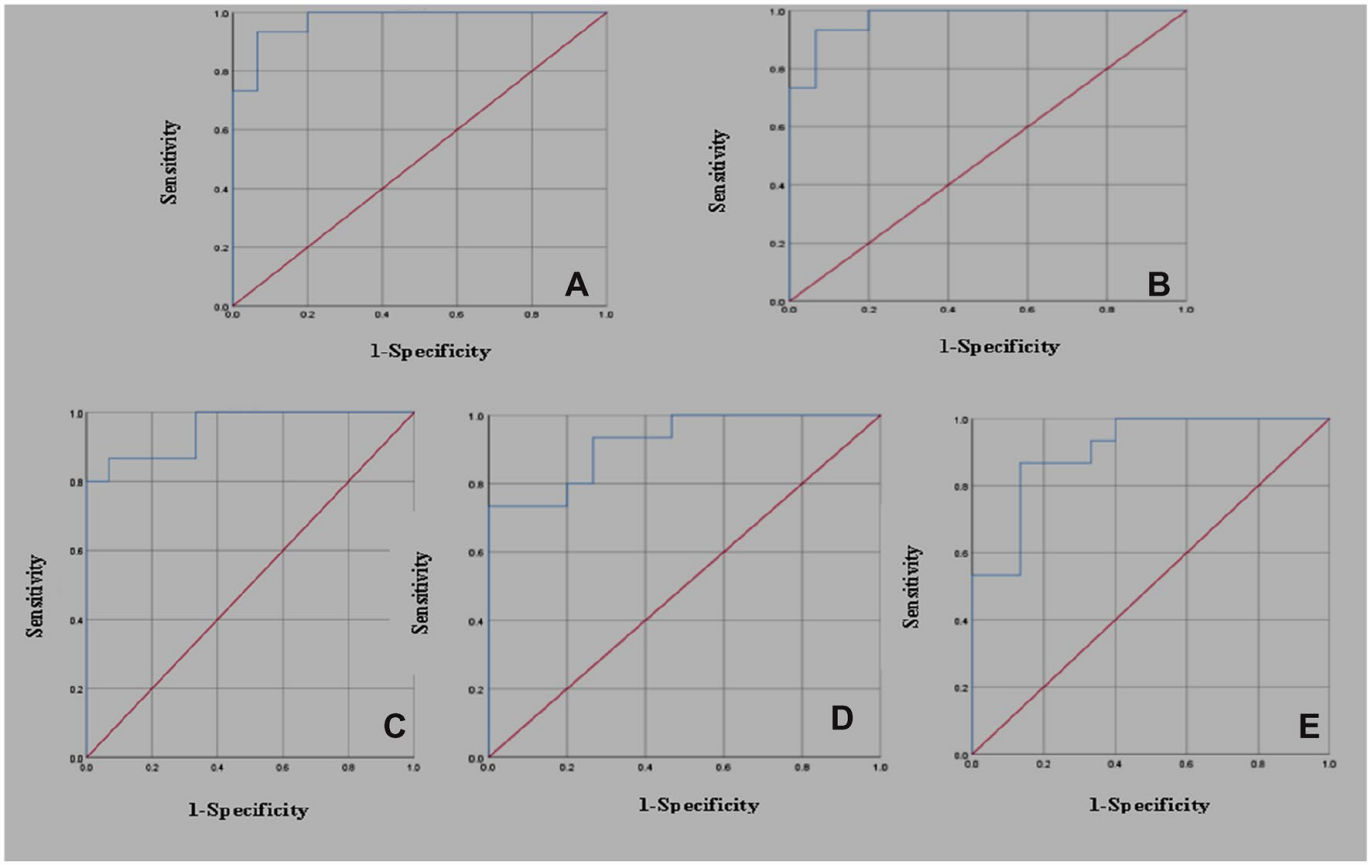
**a** Receiver operator characteristic (ROC) curve of the expression level of circ-ANAPC7 in control group/very low group. **b** ROC curve of the expression level of circ-ANAPC7 in very low group/low group. **c** ROC curve of the expression level of circ-ANAPC7 in low group/intermediate group. **d** ROC curve of the expression level of circ-ANAPC7 in intermediate group/high group. **e** ROC curve of the expression level of circ-ANAPC7 in high group/very high group

We evaluated the diagnostic value of circ-ANAPC7 expression for each risk stratification of MDS by ROC curves. The data showed that circ-ANAPC7 was able to differentiate very high-risk patients from control patients (area under the curve (AUC) = 0.973) at the best cut-off point of 12.16 (sensitivity 93.3%, specificity 93.3%); to differentiate low-risk patients from very low-risk patients (AUC = 0.996) at the best cut-off point of 43.56 (sensitivity 100%, specificity 93.3%); to differentiate intermediate-risk patients from low-risk patients (AUC = 0.951) at the best cut-off point of 149.12 (sensitivity 86.7%,specificity 93.3%); to differentiate high-risk patients from intermediate-risk patients (AUC = 0.920) at the best cut-off point of 279.25 (sensitivity 73.3%, specificity 100%); and to differentiate very high-risk patients from high-risk patients (AUC = 0.907) at the best cut-off point of 399.02 (sensitivity 86.7%, specificity 86.7%) (Table 2; Fig. 2).

**Table 2:** Area under the curve of the expression level of circ-ANAPC7

	AUC	SE	*p*	95% CI	Cut-off value
Control group/very low group	0.973	0.024	< 0.001	0.926–1	12.16
Very low group/low group	0.996	0.007	< 0.001	0.981–1	43.56
Low group/intermediate group	0.951	0.036	< 0.001	0.880–1	149.11
Intermediate group/high group	0.920	0.048	< 0.001	0.826–1	279.25
High group/very high group	0.907	0.053	< 0.001	0.802–1	399.01

## Discussion

It has become necessary to provide prognostic scores to patients with MDS in order to personalize their treatment plans. Among the scoring systems, IPSS is commonly used both to predict outcomes and to tailor therapy. Recently, the IPSS was revised to improve predictability, and the IPSS-R was developed as a result [15]. However, the prognosis is not a one-off assessment but a dynamic process that should be changed according to the treatment of the disease. The ideal scoring system should be dynamic and can be applied at different time points in the disease process. Therefore, it is imperative to find a biomarker that can dynamically evaluate risk stratification.

Numerous studies have demonstrated that ncRNAs, including circRNAs, play a crucial role in leukemia development [16–18]. Thus, AML can be effectively treated with circRNA-based therapy [19]. Researchers have discovered that circ-ANAPC7 might contribute to the pathogenesis of AML as a promising biomarker [20]. Studies show that MDS and secondary AML cells exhibit similar mutations in many genes and functional categories, confirming that they are a disease continuum [14, 21]. However, The potential role of circ-ANAPC7 in MDS has only been reported in a few studies. Hence, in our study, we explored its diagnostic value in MDS.

QRT-PCR was conducted to measure the expression level of circ-ANAPC7. The results showed that circ-ANAPC7 was significantly upregulated in MDS and gradually increased with the risk stratification of MDS, indicating that it could serve as a biomarker for MDS diagnosis. The expression level of circ-ANAPC7 showed a better performance in each risk stratification group of MDS. Furthermore, our study found that an expression level of circ-ANAPC7 > 12.16 was mighty correlated with a very low risk of MDS; > 43.56 was mighty correlated with a low risk of MDS; > 146.12 was mighty correlated with an intermediate risk of MDS; > 279.25 was mighty correlated with a high risk of MDS; and > 399.02 was mighty correlated with a very high risk of MDS.

In conclusion, the results of our study showed the significant diagnostic value of the expression level of circ-ANAPC7 as a promising biomarker for MDS. It may be a useful addition to the scoring system to better identify risk groups. However, it should be noted that this study had the limitation of a relatively small sample size. Furthermore, the ability to use circ-ANAPC7 as a potential biomarker for diagnosing and evaluating each risk stratification of MDS requires prospective studies with larger sample sizes.
